# Respiratory virus detection in the upper respiratory tract of asymptomatic, community-dwelling older people

**DOI:** 10.1186/s12879-022-07355-w

**Published:** 2022-04-28

**Authors:** Ikkoh Yasuda, Motoi Suzuki, Haruka Maeda, Mayumi Terada, Eiichiro Sando, Chris Fook Sheng Ng, Hirono Otomaru, Lay-Myint Yoshida, Konosuke Morimoto

**Affiliations:** 1 Department of Clinical Medicine, Institute of Tropical Medicine, Nagasaki University, Nagasaki, Japan; 2 Department of General Internal Medicine and Clinical Infectious Diseases, Fukushima Medical University, Fukushima, Japan; 3 Department of General Internal Medicine and Infectious Diseases, Kita-Fukushima Medical Center, Fukushima, Japan; 4 Infectious Disease Surveillance Center, National Institute of Infectious Diseases, Tokyo, Japan; 5 Department of Respiratory Infections, Institute of Tropical Medicine, Nagasaki University, Nagasaki, Japan; 6 Nijigaoka Hospital, Nagasaki, Japan; 7 Department of Global Health Policy, Graduate School of Medicine, The University of Tokyo, Tokyo, Japan; 8 School of Tropical Medicine and Global Health, Nagasaki University, Nagasaki, Japan; 9 Department of Pediatric Infectious Diseases, Institute of Tropical Medicine, Nagasaki University, Nagasaki, Japan

**Keywords:** Prevalence, Respiratory virus, Polymerase chain reaction, Asymptomatic, Community-dwelling, Older people

## Abstract

**Background:**

The prevalence of virus positivity in the upper respiratory tract of asymptomatic community-dwelling older people remains elusive. Our objective was to investigate the prevalence of respiratory virus PCR positivity in asymptomatic community-dwelling older people using saliva samples and nasopharyngeal and oropharyngeal swabs.

**Methods:**

We analyzed 504 community-dwelling adults aged ≥ 65 years who were ambulatory and enrolled in a cross-sectional study conducted from February to December 2018 in Nagasaki city, Japan. Fourteen respiratory viruses were identified in saliva, nasopharyngeal and oropharyngeal samples using multiplex PCR assays.

**Results:**

The prevalences of PCR positivity for rhinovirus, influenza A, enterovirus and any respiratory virus were 12.9% (95% CI: 10.1–16.1%), 7.1% (95% CI: 5.1–9.8%), 6.9% (95% CI: 4.9–9.5%) and 25.2% (95% CI: 21.5–29.2%), respectively. Rhinovirus was detected in 21.5% of subjects, influenza A in 38.9% of subjects, enterovirus in 51.4% of subjects and any virus in 32.3% of subjects using only saliva sampling.

**Conclusions:**

The prevalences of several respiratory viruses were higher than the percentages reported previously in pharyngeal samples from younger adults. Saliva sampling is a potentially useful method for respiratory virus detection in asymptomatic populations.

**Supplementary information:**

The online version contains supplementary material available at 10.1186/s12879-022-07355-w.

## Background

The implementation of polymerase chain reaction (PCR) in wider clinical settings facilitates the prompt and accurate detection of respiratory viruses, revealing respiratory viruses as common pathogens of community-acquired pneumonia [1–3]. A few studies have reported the prevalence of virus positivity in the upper respiratory tract of asymptomatic subjects [4–6]; however, the prevalence in community-dwelling older people susceptible to severe illness when infected has yet to be investigated.

Respiratory viral detection in the nasopharynx using molecular biological methods is a standard method to detect viral respiratory infections [7]. However, saliva specimens are potential alternative samples; saliva sampling is less invasive and is associated with a lower risk of transmission than nasopharyngeal (NP) swab sampling. The benefit of saliva sampling for PCR-based virus detection has been reported in patients infected with common respiratory viruses and severe acute respiratory syndrome coronavirus 2 (SARS-CoV-2) [8–12].

The primary objective of this study was to investigate the prevalence of PCR detection of respiratory viruses in asymptomatic community-dwelling older people in Japan. The secondary objective was to explore the possible use of saliva in addition to pharyngeal sampling for prevalence surveillance in asymptomatic populations.

## Methods

This analysis was implemented under the following existing study: “The low carriage prevalence of pneumococcus among community-dwelling older people: A cross-sectional study in Japan” [13]. All methods were conducted in accordance with the Declaration of Helsinki and the study was approved by the Ethical Review Board of the Institute of Tropical Medicine, Nagasaki University, Nagasaki, Japan, and the institutional review boards of each study facility. Written informed consent was obtained from all participants or their families. The study was conducted from February 2018 to December 2018 in Nagasaki city, Japan. We included community-dwelling older people aged ≥ 65 years who were ambulatory and attended regular clinic visits or outpatient rehabilitation visits at 4 hospitals in Nagasaki city, Japan. We excluded people with fever or any symptoms of upper respiratory tract infection, people who received antibiotic treatment in the previous 30 days, and people who were admitted to a hospital or a long-term care facility for ≥ 7 days in the previous 30 days. The detailed demographic and clinical information of the 504 participants was described previously [13]. NP and oropharyngeal (OP) samples were obtained using two swabs: one sample from the nasopharynx using a sterilized swab with an aluminum shaft (TE2201) (Eiken Chemical Co., Tokyo, Japan) and another sample from the oropharynx using a sterilized swab with a wooden shaft (TE8201) (Eiken Chemical Co., Tokyo, Japan). These swabs were immediately individually placed in 1 ml of skim milk-tryptone-glucose-glycerol (STGG) media [14]. Participants were asked to spit onto the inside of a sterilized sputum container (DE2000) (Eiken Chemical Co., Tokyo, Japan) to collect pure saliva without sputum. The details of use of these sterilized swabs and sputum containers are available online from the manufacturer [15]. Samples were collected by researchers or trained research nurses. Viral nucleic acids were extracted using a QIAamp viral RNA Mini Kit (QIAGEN Inc., Valencia, CA, USA) and QIAamp DNA Mini Kit (QIAGEN Inc., Valencia, CA, USA), and the following fourteen respiratory viruses were screened with multiplex PCR assays using a One Step RT-PCR Kit (QIAGEN Inc., Valencia, CA, USA) for RNA viruses and GoTaq Flexi DNA Polymerase (Promega, San Luis Obispo, CA, USA) and PCR Nucleotide Mix (Promega, San Luis Obispo, CA, USA) for DNA viruses, as described previously [16]: influenza A, influenza B, respiratory syncytial virus (RSV), human metapneumovirus (hMPV), parainfluenza virus types 1–4 (PIV-1, PIV-2, PIV-3 and PIV-4), rhinovirus, coronavirus 229E, OC43 (common human coronavirus [HCoV]), adenovirus, bocavirus, and enterovirus. An Additional file 1 shows the sensitivity (detection limit) of the multiplex PCR [see Additional file 1]. The prevalence of PCR positivity for respiratory viruses was defined as the total prevalence detected in at least one NP, OP and/or saliva sample. The calculation of the prevalence of PCR positivity is shown in Additional file 2: Fig. S1. Cohen’s kappa coefficient (**κ)** was computed to measure the agreement between each pair of sampling sites.

## Results

A total of 504 participants were enrolled. The median age of the participants was 77.0 years (interquartile range (IQR): 70.0, 83.0), and 257 (51.0%) were female. A total of 488 (96.8%) subjects had underlying disorders. A saliva sample could not be collected from one participant, and the saliva result was considered negative when calculating the prevalence of PCR positivity for viruses.

### The prevalence of each respiratory virus detected in each sampling site using PCR

The number of virus-positive participants and the prevalence of each respiratory virus are shown in Table 1. A total of 65 participants were positive for rhinovirus (12.9%, 95% confidence interval (95% CI): 10.1–16.1%), 36 participants were positive for influenza A (7.1%, 95% CI: 5.1–9.8%), 35 participants were positive for enterovirus (6.9%, 95% CI: 4.9–9.5%) and 127 participants were positive for any respiratory virus (25.2%, 95% CI: 21.5–29.2%). The numbers of participants who were positive for rhinovirus in NP, OP and saliva samples were 26 (5.2%, 95% CI: 3.4–7.5%), 31 (6.2%, 95% CI: 4.2–8.6%) and 33 (6.5%, 95% CI: 4.5–9.1%), respectively. The numbers of participants who were positive for influenza A in NP, OP and saliva samples were 12 (2.4%, 95% CI: 1.2–4.1%), 13 (2.6%, 95% CI: 1.4–4.4%) and 19 (3.8%, 95% CI: 2.3–5.8%), respectively. The numbers of participants who were positive for enterovirus in NP, OP and saliva samples were 13 (2.6%, 95% CI: 1.4–4.4%), 11 (2.2%, 95% CI: 1.1–3.9%) and 25 (5.0%, 95% CI: 3.2–7.2%), respectively. The numbers of participants who were positive for any virus in NP, OP and saliva samples were 47 (9.3%, 95% CI: 6.9–12.2%), 56 (11.1%, 95% CI: 8.5–14.2%) and 74 (14.7%, 95% CI: 11.7–18.1%), respectively. Additional file 3: Fig. S2 shows monthly detection of rhinoviruses, influenza A viruses, and enteroviruses from February to December 2018.

**Table 1:** The prevalence of each respiratory virus detected in each sampling site using PCR

	NP	(n = 504)	OP	(n = 504)	Saliva	(n = 504)*	Prevalence	(n = 504)
	n	% (95% CI)	n	% (95% CI)	n	% (95% CI)	n	% (95% CI)
Rhinovirus	26	5.2 (3.4–7.5)	31	6.2 (4.2–8.6)	33	6.5 (4.5–9.1)	65	12.9 (10.1–16.1)
Influenza A	12	2.4 (1.2–4.1)	13	2.6 (1.4–4.4)	19	3.8 (2.3–5.8)	36	7.1 (5.1–9.8)
Enterovirus	13	2.6 (1.4–4.4)	11	2.2 (1.1–3.9)	25	5.0 (3.2–7.2)	35	6.9 (4.9–9.5)
RSV	3	0.6 (0.1–1.7)	3	0.6 (0.1–1.7)	2	0.4 (0.0–1.4)	8	1.6 (0.7–3.1)
hMPV	0	0.0 (0.0–0.7)	4	0.8 (0.2–2.0)	2	0.4 (0.0–1.4)	6	1.2 (0.4–2.6)
HCoV	1	0.2 (0.0–1.1)	1	0.2 (0.0–1.1)	1	0.2 (0.0–1.1)	1	0.2 (0.0–1.1)
Influenza B	0	0.0 (0.0–0.7)	0	0.0 (0.0–0.7)	1	0.2 (0.0–1.1)	1	0.2 (0.0–1.1)
PIV–2	0	0.0 (0.0–0.7)	0	0.0 (0.0–0.7)	1	0.2 (0.0–1.1)	1	0.2 (0.0–1.1)
PIV–3	1	0.2 (0.0–1.1)	0	0.0 (0.0–0.7)	0	0.0 (0.0–0.7)	1	0.2 (0.0–1.1)
Any virus	47	9.3 (6.9–12.2)	56	11.1 (8.5–14.2)	74	14.7 (11.7–18.1)	127	25.2 (21.5–29.2)

*A saliva sample could not be collected from one participant, and the saliva result was considered negative when calculating the prevalence of virus positivity. *NP* nasopharyngeal, *OP* oropharyngeal, *Prevalence* the prevalence of PCR positivity, *95% CI* 95% confidence interval, *RSV* respiratory syncytial virus, *hMPV* human metapneumovirus, *HCoV* common human coronavirus, *PIV-2* parainfluenza virus type 2, *PIV-3* parainfluenza virus type 3

### The distribution of positive samples for each respiratory virus

**Fig. 1:**
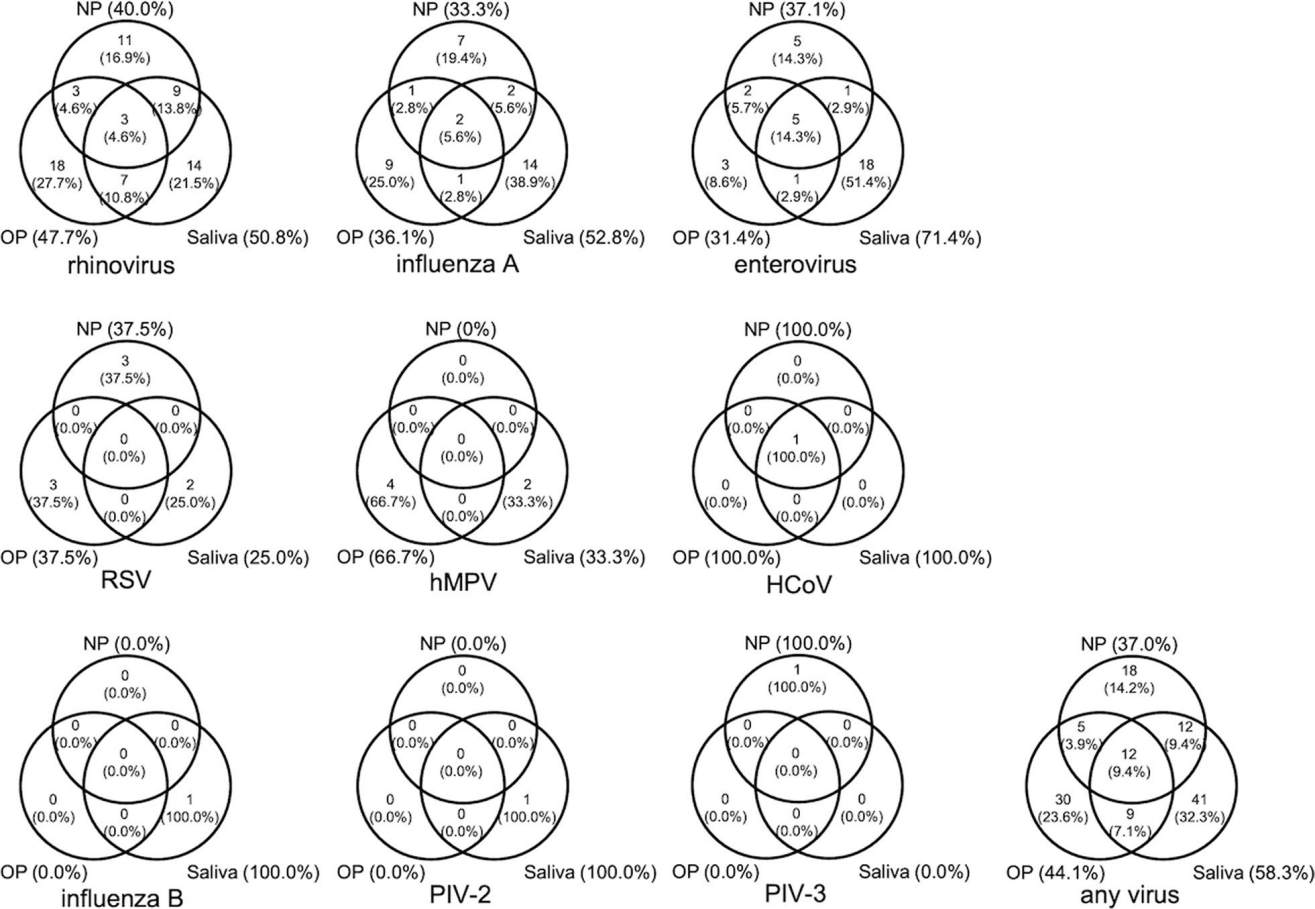
The distribution of positive samples for each respiratory virus. Each circle represents the sampling site: nasopharyngeal (NP), oropharyngeal (OP) and saliva. Numbers and percentages show the numbers and proportions of participants positive for the virus at the corresponding sampling sites. The percentage next to the sampling site indicates the proportion covered by each sampling site

Figure 1 shows the distribution of the positive samples across the three sampling sites. A low concordance of viral positivity was observed between saliva and pharyngeal samples; only 4.6% of rhinovirus-positive subjects, 5.6% of influenza A-positive subjects, 14.3% of enterovirus-positive subjects and 9.4% of any virus-positive subjects were positive at all three sampling sites. Moreover, saliva sampling enabled the detection of viruses even in subjects who were negative according to results from either NP or OP samples; 21.5% of rhinovirus-positive subjects, 38.9% of influenza A-positive subjects, 51.4% of enterovirus-positive subjects and 32.3% of any virus-positive subjects were detected by only saliva sampling. The detection in saliva samples covered 50.8% of rhinovirus-positive subjects, 52.8% of influenza A-positive subjects, 71.4% of enterovirus-positive subjects and 58.3% of any virus-positive subjects.

### Agreement among sampling sites results based on detected viruses

**Fig. 2:**
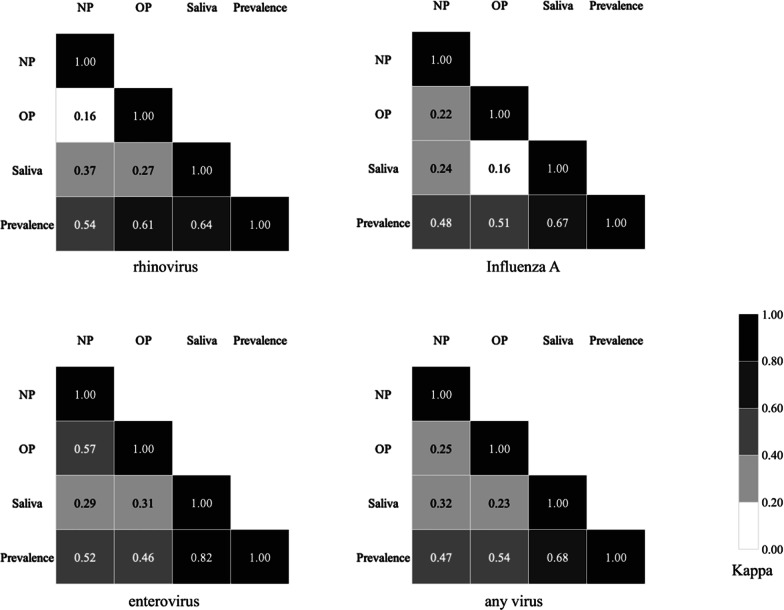
Agreement among sampling sites results based on detected viruses. Kappa coefficients represent the agreement of results between each pair of sampling sites according to the viruses detected: rhinovirus, influenza A, enterovirus and any respiratory virus. A kappa value of 1 indicates perfect agreement, while a kappa value of 0 indicates no agreement beyond chance. Darker shades indicate stronger agreement of results between sampling sites. *NP* nasopharyngeal, *OP* oropharyngeal, *Prevalence* the prevalence of PCR positivity

The degree of agreement, indicated by the kappa value, among each sampling site stratified by the type of virus detected are shown in Fig. 2. The kappa values between saliva and pharyngeal samples ranged from 0.16 (between saliva and OP samples for influenza A) to 0.37 (between saliva and NP samples for rhinovirus).

## Discussion

This study documented the prevalences of PCR positivity for rhinovirus, influenza A, enterovirus and any respiratory virus, which were higher than the percentages in asymptomatic younger adults reported previously with NP and/or OP sampling. A study from Israel reported a 2.0% prevalence of rhinovirus, a 0.4% prevalence of influenza A and a 7.1% prevalence of any respiratory virus detected using real-time PCR in an ambulatory group aged > 18 years without fever or respiratory symptoms using NP swabs, OP swabs and NP washing [4]. A 0.8% prevalence of rhinovirus, a 0.0% prevalence of influenza A and a 2.1% prevalence of any respiratory virus were reported in an asymptomatic group aged ≥ 18 years from the U.S. using real-time PCR with NP and OP swabs [17]. Another study from Sweden reported a 3.2% prevalence of rhinovirus, a 0.0% prevalence of influenza A, a 0.2% prevalence of enterovirus and a 4.3% prevalence of any respiratory virus detected using real-time PCR in a group aged ≥ 18 years without symptoms consistent with respiratory tract infections using NP swab samples [6]. The higher viral prevalences in asymptomatic community-dwelling older adults suggest the significance of viral existence in the upper respiratory tract that may serve as a reservoir for respiratory viruses, a source of transmission between hosts or an induction of disease development.

This study demonstrated the possible use of saliva in addition to pharyngeal sampling for prevalence surveillance. In Table 1, the estimated 95% CIs for the detection of rhinovirus, influenza A and enterovirus largely overlapped between the NP, OP and saliva sampling sites, suggesting comparable value of the sampling sites for the detection of these viruses. However, when all the sampling methods were combined, the estimated prevalences were generally higher, particularly compared to the results of NP and OP sampling alone, for which the 95% CIs were consistently lower for the detection of these viruses. Further analysis showed that the level of agreement between saliva and pharyngeal samples was not high (kappa < 0.4), as seen in Fig. 2. These results suggest the potential of saliva sampling to detect certain cases missed by pharyngeal sampling alone, notably for influenza A and enterovirus, for which saliva sampling alone detected more than a third of the total positive cases (38.9% and 51.4% respectively in Fig. 1). Although saliva sampling does not replace pharyngeal sampling because it missed some cases detected by pharyngeal sampling, the findings indicate that adding saliva sampling to pharyngeal sampling can increase the detection rate of several respiratory viruses and that saliva sampling alone could be an alternative option to approximate prevalence and trends in specific settings where pharyngeal sampling is not practical. However, the results must be interpreted with caution because of the low concordance of viral positivity between saliva and pharyngeal samples. A limited number of studies have evaluated the utility of saliva for respiratory virus detection in adults. In a study from Korea, the performance of saliva samples was reported to be equivalent to that of NP swabs for the detection of 16 respiratory viruses in adult male patients with suspected acute respiratory illnesses using multiplex real-time PCR assays [12]. A study from China showed the potential use of saliva samples in addition to nasopharyngeal aspirates to improve the detection of respiratory viruses by multiplex PCR in hospitalized adult patients with suspected respiratory infections [18]. Another study reported the reliability of saliva sampling for respiratory virus testing with point-of-care molecular assays in adult hospitalized patients with respiratory tract infection [19]. During the coronavirus disease 2019 (COVID-19) pandemic, further insights into saliva sampling have been suggested. The role of saliva in viral transmission has been emphasized, and some studies compared the viral loads in upper respiratory samples obtained from multiple sampling sites in patients infected with SARS-CoV-2 and suggested that the viral load at each sampling site might relate to the clinical background, such as severity, transmissibility, phase of the disease and presence or absence of symptoms [20–22]. On the other hand, a low sensitivity of saliva sampling in asymptomatic SARS-CoV-2 carriers was reported, and the utility of saliva in asymptomatic populations remains controversial [23]. The pure saliva sampling method that was used in this study can be implemented in clinical practice and surveillance because of its practicality for people who lack sputum production, and it does not require special collection devices or expertise. However, this method may not always be feasible, especially in older people who may have difficulty expelling a sufficient volume of saliva, as observed in the present study and other studies [19].

Our study has several limitations. This cross-sectional study lacked longitudinal data, and we were unable to determine if the positive test results represented participants who were asymptomatic or presymptomatic. While this study included results obtained from multiple sites, all were from one city, and further data must be accumulated from various countries and clinical settings to generalize our findings. Although the sensitivity of our multiplex PCRs was technically estimated using serial diluted PCR products in vitro, the clinical sensitivity might vary based on the type of samples (NP, OP or saliva). The viral loads were not able to be evaluated because conventional PCR was performed and quantitative data were not available, and our results might be influenced by the sensitivity of the assay used in the current study. Thus, further work is needed to assess the viral loads and their clinical significance. Because our assay lacked sequencing, we could not differentiate strictly between human rhinoviruses and human enteroviruses. Selection bias might have been introduced because we enrolled the participants at regular clinic visits or outpatient rehabilitation visits, and this population tends to have a greater need for care and more comorbidities than healthy individuals. We did not clearly determine whether saliva was useful for the detection of pathogens with low prevalence in this study, namely, viruses other than rhinovirus, influenza A and enterovirus.

## Conclusions

This study describes the prevalence of the PCR detection of respiratory viruses in asymptomatic community-dwelling older people using saliva in addition to NP and OP samples. The prevalence of some viruses was higher than that previously reported in younger adults using pharyngeal samples. Although the study showed the potential of saliva for viral detection in asymptomatic populations, further studies are warranted to verify the utility of saliva and the clinical significance of the low concordance of viral positivity between saliva and pharyngeal samples.

## Supplementary information

**Additional file 1. **The sensitivity of the multiplex PCR.

**Additional file 2: Fig. S1. **The calculation of the prevalence of PCR positivity.

**Additional file 3: Fig. S2.** Monthly detection of rhinoviruses, influenza A viruses, and enteroviruses from February to December 2018.

## Data Availability

The datasets used and/or analyzed during the current study are available from the corresponding author upon reasonable request.

## Abbreviations

COVID-19: Coronavirus disease 2019
HCoV: Common human coronavirus
hMPV: Human metapneumovirus
IQR: Interquartile range
NP: Nasopharyngeal
OP: Oropharyngeal
PCR: Polymerase chain reaction
PIV-1, PIV-2, PIV-3 and PIV-4: Parainfluenza virus types 1–4
RSV: Respiratory syncytial virus
SARS-CoV-2: Severe acute respiratory syndrome coronavirus 2
STGG: Skim milk-tryptone-glucose-glycerol
95% CI: 95% confidence interval
