# Supplementary material for: Respiratory virus detection in the upper respiratory tract of asymptomatic, community-dwelling older people
Source: BMC Infect Dis. 2022 Apr 28;22:411. doi: 10.1186/s12879-022-07355-w (PMC9047617; doi:10.1186/s12879-022-07355-w)
Supplement: Supplementary file 1 — Additional file 1. The sensitivity of the multiplex PCR. [file 12879_2022_7355_MOESM1_ESM.docx]

**Additional file 1 The sensitivity of the multiplex PCR**

We conducted the following assays to obtain approximate estimates of the detection limit in 13 virus multiplex PCRs. First, the targeted fragments were amplified by PCR and confirmed by 2% agarose gel electrophoresis. The PCR products were then extracted from the gel with a QIAquick Gel Extraction Kit (QIAGEN, Germany). A Qubit ® dsDNA HS assay kit and Qubit 4 Fluorometer (Thermo Fisher Scientific, MA, USA) were used to quantify the double-strand DNA concentration. The double-strand DNA copy number was calculated for each PCR product. Three sets of 10-fold serial diluted PCR products from 1:10 to 1: 10^11^ were prepared, and each dilution was tested by multiplex PCR followed by 2% agarose gel electrophoresis. PCR for three dilution sets was conducted in duplicate, and the detection limit (copies/µl) was determined by the concentration at which the band in electrophoresis was visible in all PCR assays. The following table summarizes the results.

| PCR assay | Target virus | Approximate detection limit (copies/µl) |
| --- | --- | --- |
| mPCR1 | Influenza virus A | <10 |
|  | Influenza virus B | <10 |
|  | Respiratory Syncytial Virus | <10 |
|  | Human metapneumovirus | <10 |
| mPCR2 | Parainfluenza virus 1 | <10000 |
|  | Parainfluenza virus 2 | <1000 |
|  | Parainfluenza virus 3 | <1000 |
|  | Parainfluenza virus 4 | <100000 |
| mPCR3 | Rhinovirus | <10 |
|  | Human coronavirus 229E | <100 |
|  | Human coronavirus OC43 | <10 |
| mPCR4 | Adenovirus | <10 |
|  | Bocavirus | <10 |

Note: The sensitivity of the assay was low for PIV4. The reason for this is that we did not have the PIV4 control when we set up the assay, so we used a set of published primers in 2000 for the assay. The control that we used in the current detection limit assay was from PIV4-positive clinical samples that have some polymorphism in the primer binding site, which led to poor sensitivity. Regarding the primers DK001 and DK004 used for enterovirus PCR assay in the current study and the details, please refer to the papers below.

PIV4 primer reference paper

Aguilar JC, Perez-Brena MP, Garcia ML, Cruz N, Erdman DD, Echevarria JE: Detection and identification of human parainfluenza viruses 1, 2, 3, and 4 in clinical samples of pediatric patients by multiplex reverse transcription-PCR. *J Clin Microbiol* 2000, 38(3):1191-1195.

Enterovirus primer reference papers

Kiang D, Yagi S, Kantardjieff KA, Kim EJ, Louie JK, Schnurr DP: Molecular characterization of a variant rhinovirus from an outbreak associated with uncommonly high mortality. *J Clin Virol* 2007, 38(3):227-237.

Kiang D, Kalra I, Yagi S, Louie JK, Boushey H, Boothby J, Schnurr DP: Assay for 5' noncoding region analysis of all human rhinovirus prototype strains. J Clin Microbiol 2008, 46(11):3736-3745.

Fig. Example gel image of the influenza A detection limit
