# Supplementary material for: Respiratory virus detection in the upper respiratory tract of asymptomatic, community-dwelling older people
Source: BMC Infect Dis. 2022 Apr 28;22:411. doi: 10.1186/s12879-022-07355-w (PMC9047617; doi:10.1186/s12879-022-07355-w)
Supplement: Supplementary file 2 — Additional file 2: Fig. S1. The calculation of the prevalence of PCR positivity. [file 12879_2022_7355_MOESM2_ESM.docx]

**

**

**Additional file 2: Fig. S1 The calculation of the prevalence of PCR positivity**

The calculation for the prevalence of PCR positivity is shown in the figure. Each circle represents the sample site: nasopharyngeal (NP), oropharyngeal (OP) and saliva. Each alphabet shows the number of participants positive for the corresponding sample sites.
