# Supplementary material for: Respiratory virus detection in the upper respiratory tract of asymptomatic, community-dwelling older people
Source: BMC Infect Dis. 2022 Apr 28;22:411. doi: 10.1186/s12879-022-07355-w (PMC9047617; doi:10.1186/s12879-022-07355-w)
Supplement: Supplementary file 3 — Additional file 3: Fig. S2. Monthly detection of rhinoviruses, influenza A viruses, and enteroviruses from February to December 2018. [file 12879_2022_7355_MOESM3_ESM.docx]

**
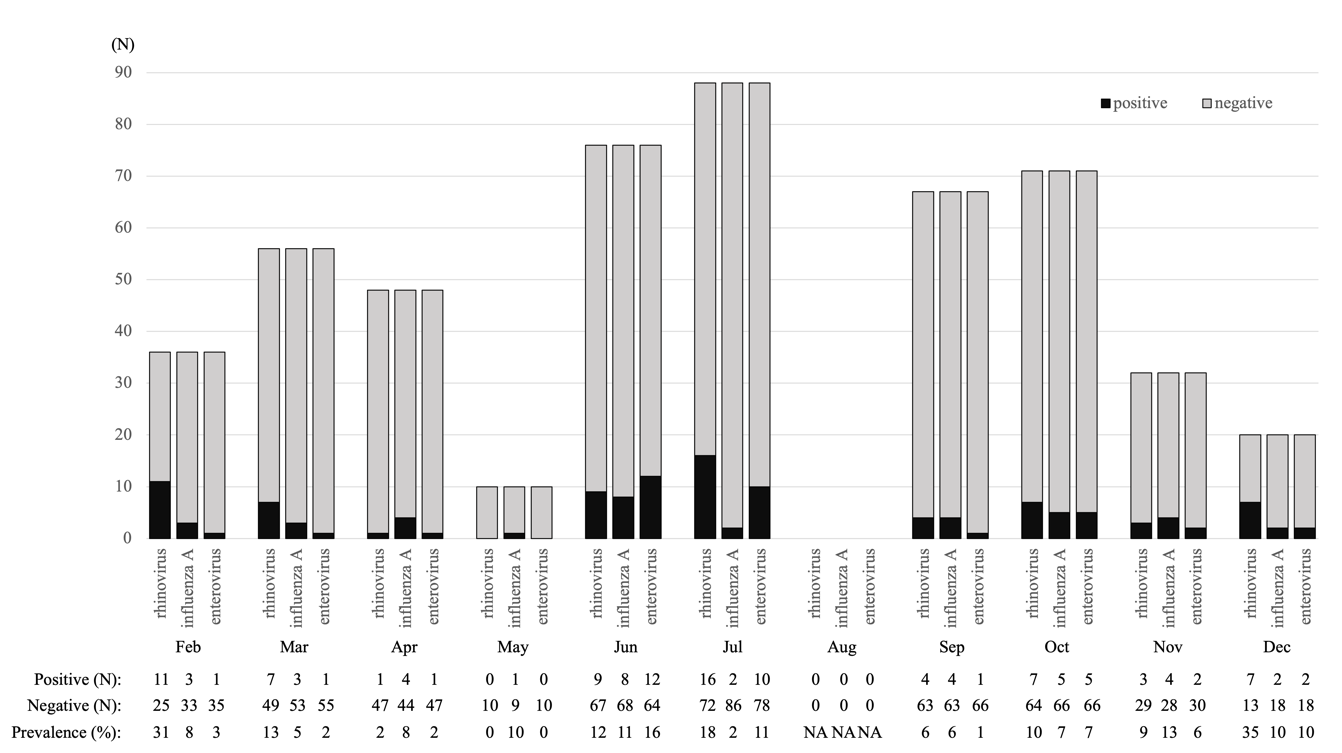
**

**Additional file 3: Fig. S2 Monthly detection of rhinoviruses, influenza A viruses, and enteroviruses from February to December 2018.**

The numbers of participants positive and negative for rhinovirus, influenza A and enterovirus are shown in black and gray, respectively. Positive (N) and Negative (N) show the actual numbers of participants positive and negative for each virus, respectively. Prevalence (%) shows the prevalence of viruses detected by PCR by month of collection. “The prevalence of viruses detected by PCR” was defined as the prevalence detected from at least one NP, OP and/or saliva sample. One saliva sample could not be collected from one participant, and the saliva result was considered negative in calculating the prevalence of virus positivity. NA: not applicable.
